# Administration of ketoprofen in postpartum sows to control the incidence of post-parturient disorders and improve piglet survival rate

**DOI:** 10.5713/ab.22.0392

**Published:** 2023-05-02

**Authors:** Suwicha Jeeraphokhakul, Thanabat Theerakulpisut, Pitchapa Khampoomee, Jakkrit Chaiwangna, Preechaphon Taechamaeteekul, Natchanon Dumniem, Junpen Suwimonteerabutr, Padet Tummaruk

**Affiliations:** 1 Department of Obstetrics, Gynaecology and Reproduction, Faculty of Veterinary Science, Chulalongkorn University, Bangkok 10330, Thailand; 2 Centre of Excellence in Swine Reproduction, Chulalongkorn University, Bangkok 10330, Thailand

**Keywords:** Colostrum, Inflammation, Ketoprofen, Lactational Pig

## Abstract

**Objective:**

Inflammation and pain management in postpartum hyperprolific sows is currently an important animal welfare issue in the swine industry. The present study investigates effects of ketoprofen treatment on the incidence of post-parturient disorders, feed intake, colostrum yield, piglet colostrum intake, colostrum immunoglobulin G (IgG) and piglet mortality rate during the first 3 days of postnatal life.

**Methods:**

In total, 61 Danish Landrace×Yorkshire crossbred sows and their offspring (n = 833) were included in the experiment. The sows were randomly distributed into two groups: i) control (n = 31), sows were treated with tolfenamic acid 2 mg per kg for 2 days postpartum; ii) ketoprofen (n = 30), sows were treated with ketoprofen 3 mg per kg for 2 days postpartum. The farrowing process of the sows was monitored for 24 h daily, and data associated with farrowing were collected. Piglet colostrum intake, sow colostrum yield and colostrum IgG were determined.

**Results:**

During the first 3 days postpartum, the incidence of sows that had fever did not differ between control and ketoprofen groups (51.6% and 56.7%, respectively, p = 0.692). Piglet colostrum intake did not differ between control and ketoprofen groups (p = 0.736). However, the proportions of piglets that had inadequate colostrum intake were 71.3%, 22.6%, and 5.4% in those with birth weights of <1.0 kg, 1.0 to 1.29 kg, and ≥1.30 kg, respectively (p<0.001). The piglet mortality rate did not differ between control and ketoprofen groups (p = 0.808).

**Conclusion:**

Administration of ketoprofen in postpartum sows for 2 days can control the evidence of post-parturient disorders in sows as effectively as the use of tolfenamic acid. No deleterious effect of ketoprofen was detected on sow colostrum yield, piglet colostrum intake and piglet mortality. Therefore, ketoprofen can be recommended as an alternative anti-inflammatory drug used in postpartum sows.

## INTRODUCTION

Inflammation and pain management in postpartum hyperprolific sows is currently an important animal welfare issue in the swine industry [1]. Prolonged parturition is one of the most important factors causing moderate to severe inflammation and pain in hyperprolific sows [2,3]. As a consequence, both pre- and post-farrowing management measures are becoming interesting research areas to be explored. Earlier studies have demonstrated that the long duration of farrowing in hyperprolific sows goes along with a risk of increasing the incidence of post-parturient disorders in sows and can compromise colostrum intake of newborn piglets [4,5]. Vongsariyavanich et al [5] have demonstrated that the piglets born during the first half of the farrowing process had a significantly higher colostrum intake than those born during the second half of the farrowing process. Tummaruk and Sang-Gassanee [4] found that the incidence of sows with fever (i.e., rectal temperature ≥39.0°C) was increased from 40% to 100% when the farrowing duration increased from < 2.0 h to 4–8 h. Additionally, fever can be detected in 93.7% of primiparous sows and in 52.6% and 47.6% of sow parity 2 to 4 and 5 to 7, respectively, on the first day postpartum [4]. Moreover, farrowing problems can lead to a reduced feed intake of sows, a reduced survival rate of piglets and compromise subsequent reproductive performance [2,6]. The major causes of piglet preweaning mortality are associated with non-infectious causes and mainly due to insufficient colostrum intake [7]. Decreased colostrum production in postpartum sows is associated with inflammation of the reproductive system [6]. A long farrowing duration is related with constipation and stress [8]. Pain from parturition can compromise sow appetite and reduce daily feed intake, subsequently affecting the quantity and quality of colostrum [9]. One of the crucial components in sow colostrum is immunoglobulin, which plays an important role in piglet growth and survival [10]. Thus, inadequate immunoglobulin levels in sow colostrum can compromise the growth and survival rates of the suckling piglets. These findings indicate that inflammation and pain management should be done as soon as possible after farrowing to minimize postpartum complications in sows and enhance their colostrum quantity and quality during early lactation.

In swine industry, either meloxicam or tolfenamic acid is commonly used in post-partum sows to reduce pain and inflammation caused by the long labor period of sows [1,11]. Moreover, flunixin meglumine is also a non-steroidal anti-inflammatory drug (NSAIDs) commonly known and is occasionally used in swine [4]. In the European Union (EU), five types of NSAIDs and two NSAID-like drugs have been approved for pigs, including meloxicam, flunixin meglumine, tolfenamic acid, ketoprofen, sodium salicylic acid, as well as paracetamol and metamizole [11]. Based on a previous literature review, most of the earlier studies concerning NSAIDs focused on the efficacy of NSAID-related pain alleviation in piglet castration and in experimental inflammation models; thus, clinical research on the use of NSAIDs in sows is limited [11] and, to our knowledge, only one study on flunixin meglumine has been conducted in a tropical environment [4]. Therefore, the scientific data on the clinical use of other NSAIDs, such as tolfenamic acid and ketoprofen, in postpartum sows should be adequately addressed. Tolfenamic acid is an NSAIDs with analgesic, anti-inflammatory and antipyretic properties that is commonly used in humans, dogs and cats, and it has also been registered for use in sows with postpartum dysgalactia syndrome (PDS) [11]. However, a previous study has demonstrated that treatment with tolfenamic acid at 10 min before castration resulted in higher cortisol concentrations after castration, compared with meloxicam or ketoprofen [12]. Thus, the time of administration of tolfenamic acid can be a crucial factor in pain alleviation [11]. To our knowledge, the clinical use of tolfenamic acid has not yet been sufficiently investigated to clarify their efficacy and address the clear indications for its use in sows [11].

Ketoprofen is an NSAIDs that has analgesic, anti-inflammatory and antipyretic properties. Generally, ketoprofen medication can be applied either intramuscularly or orally. However, the concentration of ketoprofen in the blood circulation after intramuscular administration is higher compared to that after oral administration [13,14]. The recommended dose of ketoprofen for intramuscular administration is 3 mg per kg body weight [15]. Ketoprofen formulations for veterinary use are licensed as racemic compound (+) isomer [16,17]. In practice, ketoprofen is commonly used for the treatment of problematic sows and respiratory infections in pigs [18]. A previous study has demonstrated that a single injection of ketoprofen within 1.5 h after farrowing can improve piglet survival during the lactation period [19]. However, there are no clinical studies on the use ketoprofen in post-parturient sows in a tropical environment in relation to farrowing duration, sow parity number, colostrum yield and piglet survival. The objectives of the present study were to investigate the effects of postpartum ketoprofen treatment on the incidence of post-parturient disorders, feed intake, sow colostrum yield, piglet colostrum intake, colostrum immunoglobulin G (IgG) and piglet mortality rate during the first 3 days of postnatal life.

## MATERIALS AND METHODS

### Animal care

The present study followed the guidelines documented in The Ethical Principles and Guidelines for the Use of Animals for Scientific Purposes, edited by the National Research Council of Thailand, and was approved by the Institutional Animal Care and Use Committee in accordance with Chulalongkorn University regulations and policies governing the care and use of experimental animals (animal use protocol number 2031039).

### Experimental design

The present study was a case-control study including 61 Danish Landrace×Yorkshire crossbred multiparous sows and their offspring (n = 833 newborn piglets). It was conducted in a commercial swine herd in the western region of Thailand between July and August 2020. The sows were randomly distributed into two groups: i) control (n = 31), sows were treated with tolfenamic acid 2 mg per kg for 2 days postpartum; and ii) ketoprofen (n = 30), sows were treated with ketoprofen 3 mg per kg for 2 days postpartum. The farrowing process of the sows was monitored from the start to the end for 24 h daily by the research team. All records associated with farrowing were carefully collected. Postpartum sow characteristics, i.e., the occurrence of PDS, abnormal vaginal discharge, post-parturient fever and daily feed intake of sows, were determined for 4 days postpartum. Data on piglet birth weight, body weight at 24 h postpartum and piglet mortality during the first 3 days of postnatal life were collected. Piglet colostrum intake and sow colostrum yield were estimated using an equation developed by Theil et al [20]. Sow colostrum IgG was determined by using both enzyme-linked immunosorbent assay (ELISA) and Brix refractometer.

### Housing and management

Sows were kept in closed houses equipped with an evaporative cooling system and temperature control facilities to maintain optimal temperatures inside the barn. The average ambient temperature during the experimental period ranged from 25.5°C to 27.0°C. The daily minimum and maximum temperatures ranged from 23.8°C to 24.4°C and from 28.6°C to 32.0°C, respectively. Sow parity number averaged 5.5±1.6 (range, 3 to 9). Numbers of sows in parity numbers 3, 4, 5, and 6 to 9 were 6, 13, 12 and 30, respectively. The gestating sows were kept in individual crates (1.25 m^2^) and fed a commercial gestation diet twice daily (0700 h and 1500 h), with an average of 2.5 kg per sow per day [21]. Gestating sows were moved to the farrowing house at 1 week before the expected date of parturition. In the farrowing house, the sows were placed in individual crates (1.5 m^2^) placed at the center of the pens with a space allowance of 4.2 m^2^. The pens were fully slatted with concrete at the center for sows and had steel slats at both sides of the farrowing crate for piglets. The farrowing process was carefully supervised by a research team for 24 h daily. The farrowing process was defined as the interval from the expulsion of the first piglet to the last piglet born. Farrowing assistance was performed only when dystocia was clearly identified. Birth assistance, including manual extraction of the piglets and administration of 20 IU oxytocin intramuscularly (CP-CIN20; LBS Laboratory, Bangkok, Thailand), was performed when the expulsion interval exceeded 45 min. According to the herd veterinarian recommendation, all sows were medicated with antibiotics (amoxicillin trihydrate 150 mg/mL, 10 mg/kg, Amoxiguard 15% Injection LA; BIC Chemical co. ltd., Nakhon Pathom, Thailand) and NSAIDs within 1 h after the end of the farrowing process. Sows in the control group were treated with tolfenamic acid 2 mg/kg for 2 days postpartum (tolfenamic acid, 40 mg/mL, 2 mg/kg, Tolfedine CS; Vetoquinol S.A., Lure Cedex, France). Sows in the ketoprofen group were treated with ketoprofen 3 mg/kg for 2 days postpartum (ketoprofen 100 mg/mL, Ainil, Invesa Industrial Veterinaria S.A.; LIVISTO company, Barcelona, Spain). The first administration of the NSAIDs was done within 1 h postpartum, and administration was repeated within 24 h after the first administration. Two sows in the ketoprofen group and four sows in the control group were also repeatedly administered NSAIDs on the third day after farrowing due to illness. During lactation, sows were fed twice daily with a lactation diet to meet or exceed their nutritional requirements [21]. After farrowing, the amount of feed offered to sows increased daily to reach 7 kg per sow per day within 1 week postpartum. Sows and piglets had *ad libitum* access to water by one nipple for the sow and one nipple for the piglets. Routine procedures performed on piglets included weighing, tail docking, tooth clipping and administration of 1 mL (200 mg) iron supplement intramuscularly (IRON 10%; Bic Chemical Co., Ltd., Nakhon Pathom, Thailand) on the third day of life. Piglets were orally administered a coccidiocide (Toltrazuril, 50 mg/mL, 20 mg/kg, Toltrazuril 5%; Better Pharma Co., Ltd., Bangkok, Thailand) on the third day of life. Weaning took place at 23.0±2.0 days after farrowing.

### Sow data

The sow parameters collected included gestation length (day), farrowing duration (the time interval between the expulsion of the first and last piglets in hours), total number of piglets born per litter, number of piglets born alive per litter, number of stillborn piglets per litter and number of mummified fetuses per litter. Also, it was recorded whether sows required birth assistance (yes/no). The clinical signs of postpartum complications, including the presence of fever, PDS, abnormal vaginal discharge and constipation score, were determined in each sow at days 0, 1, 2, and 3 of parturition. Rectal temperature was determined twice a day (0700 h and 1900 h) in each sow with a digital thermometer (SOS plus Clinical digital thermometer BT-A21CN WHITE, SOS; DKSH Co. Ltd., Bangkok, Thailand). Sows with a rectal temperature of ≥39.5°C were regarded as having a fever [22]. Fever was a binomial trait, defined as ‘0’ when the sows had no fever and ‘1’ when the sows had a fever. Also, the characteristics of vaginal discharge were evaluated. Abnormal vaginal discharge was defined according to the presence or absence of postpartum abnormal vaginal discharge (i.e., exudates or bloody discharge of dark grey to white in color). Abnormal vaginal discharge was defined as ‘1’ when a certain amount of abnormal discharge (≥5 mL) was observed and ‘0’ if the abnormal discharge was absent or only a small amount of transudate (lochia) was seen [23]. The incidence of abnormal vaginal discharge was observed for 3 days postpartum, and the most severe clinical signs of vaginal discharge observed were used in the analyses. The PDS was defined according to the presence or absence of udder inflammation and/or agalactia; inflammation is the reddening and swelling of the udder. If at least one udder had inflammation or agalactia, PDS was defined as ‘1’; otherwise, it was defined as ‘0’ [23]. The constipation scores of each sow were ranked by a visual qualitative evaluation and defined as score values ranging from 0 to 5: ‘1’ (dry and pellet-shaped), ‘2’ (between dry and normal), ‘3’ (normal and soft, but firm and well formed), ‘4’ (between normal and wet; still formed but not firm) and ‘5’ (very wet feces, unformed and liquid) [6]. Additionally, voluntary feed intake of each individual sows was collected for 4 days postpartum. The feed was determined for each meal from the difference between feed allowance and the remaining feed (after drying) collected 1 h after the meal. Therefore, the total amount of feed intake was recorded for each sow twice daily. Feeding was provided twice daily at 0700 h and 1500 h. The daily feed intake of sows was defined as the sum of the feed intake at 0700 h and 1500 h.

### Piglet data

The piglet parameters recorded during the study consisted of birth order, birth interval (the time elapsed between each piglet born, min), birth weight and day of mortality (day). All piglets were individually identified by a pen marker on their back. Body weight of the piglets was determined immediately after birth and again at 18 to 24 h and at day 3 of postnatal life by using a digital bench scale (WEIGHT INDICATOR MI-01, Linear; Linear Instrument Ltd., Part, Nakhon Pathom, Thailand). The piglets were classified according to body weight at birth into three groups: low (<1.0 kg), moderate (1.0 to 1.29 kg) and high (>1.3 kg). The individual colostrum intake of each piglet was estimated with the equation developed by Theil et al [20]: Colostrum intake (g) = −106+2.26 WG+200 BWB+0.111 D−1414 WG/D +0.0182 WG/BWB; where CI is the colostrum intake (g), WG is the weight gain of individual piglets from birth to 24 h after the first born piglet (g), BWB is the weight of individual piglets at birth (kg), and D is the duration of colostrum intake (min). A colostrum intake of less than 300 grams was considered inadequate [24]. Additionally, the colostrum intake per kg of the piglet birth weight was also calculated: colostrum intake per kg of the piglet birth weight = colostrum intake/piglet birth weight, kg. The colostrum yield of sows was calculated by summing the colostrum intake of each individual piglet within the litter.

### Determination of colostrum immunoglobulin G

Colostrum samples (n = 61) were collected within 3 h after the first piglet was born to determine the estimated value of colostrum IgG content (Brix values) [10] shortly after collection (0.3 mL), and the remaining colostrum (5 mL) was kept at −20°C for determining colostrum IgG using ELISA. The measurement of Brix value of fresh colostrum in sows has been validated to be use as a rapid method for estimating IgG concentration of colostrum [10]. A commercial digital refractometer (digital hand-held pocket refractometer, ATAGO, Tokyo, Japan) was used, with a range of 0% to 53% Brix [10]. The colostrum sample was manually collected from all functional glands in a clean bottle and stored on ice in a Styrofoam box (4°C) during the collection process. The colostrum samples were centrifuged at 13,000×g for 20 min at 4°C (Centrifuge 5810 R; Eppendorf AG, Hamburg, Germany). Thereafter, the fat was discarded, and the remaining liquid was collected. After that, the liquid part was diluted at 1:500,000 with a sample conjugate diluent (50 mM Tris buffer, 0.14 M NaCl, 1% bovine serum albumin and 0.05% Tween 20), and the concentration of IgG was determined using ELISA. The ELISA plate was coated with the polyclonal antibody of Pig-IgG (Pig IgG ELISA kit; Bethyl Laboratories Inc., Montgomery, TX, USA), and the IgG concentration in the sow colostrum was determined according to our previous study [25]. All analyses were done in duplicated samples. The inter- and intra-assay coefficients of variation were 3.75% and 2.00%, respectively.

### Statistical analysis

The statistical analyses were performed using SAS version 9.4 (SAS Inst. Inc., Cary, NC, USA) [26]. Descriptive statistics, including number of non-missing values, general means, standard deviation (SD) and range, were conducted for all continuous data. Frequency analyses were conducted to evaluate categorical data. The data were classified into two groups, i.e., sow data (n = 61) and piglet data (n = 833). The sow data included parity number, gestation length, farrowing duration, total number of piglets born per litter, number of piglets born alive per litter, percentage of stillbirths and mummified fetuses and colostrum yield. The continuous data were analyzed using the generalized linear model procedure of SAS. The statistical models included the effects of treatment groups (control and ketoprofen), parity group (3 to 5 and 6 to 9) and two-way interaction. The sow was considered as the experimental unit. Least-square means were obtained from each class of the factors and compared using the least significant difference test.

The piglet data included birth interval, birth weight, body weight at 24 h, colostrum intake and colostrum intake per kg of the piglet birth weight; they were analyzed using the generalized linear mixed model (MIXED) procedure of SAS. The statistical models included treatment groups, parity group (3 to 5 and 6 to 9) and two-way interaction. Sow identity was included in the statistical model as a random effect to adjust for the maternal effect. Least-square means were obtained from each class of the factors and compared using the least significant difference test.

Postpartum complications indices including fever (yes, no), PDS (yes, no), abnormal vaginal discharge (yes, no) and constipation (score 0 to 5), in control and ketoprofen groups, were observed for 3 days postpartum. The incidences of PDS and abnormal vaginal discharge during the first 3 days postpartum were presented as percentages and compared between groups using Chi-square test. The incidence of fever on Days 1, 2 and 3 postpartum in control and ketoprofen groups were presented as percentage and were analyzed using the generalized linear mixed model procedure (GLIMMIX) of SAS. The statistical models included treatment groups, day postpartum (1, 2, and 3) and two-way interaction. The sow was considered the experimental unit. Least-square means were obtained from each class of the factors and compared using the least significant difference test. The average of fecal score during the first 3 days postpartum was calculated and were compared between groups using Wilcoxon rank sum test. In addition, the incidence of postpartum complication parameters was also compared between sows with a prolonged farrowing duration (≥300 min) and sows with normal farrowing duration (<300 min) by using the Chi-square test. Across groups, the associations between sow colostrum yield, colostrum IgG, Brix values, farrowing duration, rectal temperature and litter traits were analyzed using Spearman’s correlation. For all statistical tests, p<0.05 was considered statistically significant.

## RESULTS

### Descriptive data

Reproductive performance, farrowing duration, colostrum yield and post-parturient disorders in sows included in the experiment are presented in Table 1. On average, the total numbers of piglets born per litter and of piglets born alive per litter were 15.9±3.0 and 13.7±2.7, respectively. The incidences of stillbirth and mummified fetuses were 9.1% and 3.8%, respectively. Of these sows, 60.7% had a total number of piglets born per litter of ≥16, and 34.7% had numbers of piglets born alive per litter of ≥15. On average, gestation length and farrowing duration of sows were 116.2±1.2 days (range, 113 to 119) and 277.8±164.4 min (range, 54 to 948), respectively (Table 1). Of all sows, 29.5% (18/61) had a farrowing duration of longer than 300 min. Sow colostrum yield averaged 5.39±1.07 kg and varied among sows from 2.68 to 7.75 kg (Table 1). Most of the sows (85.3%) produced between 4 and 6 kg of colostrum. In both groups, most of the postpartum sows were treated with NSAIDs twice. However, two sows (6.6%) in the ketoprofen group (n = 30) and four sows (12.9%) in the control group (n = 31) required the third treatment. The proportion of sows that required farrowing assistance was 34.4% (21/61 sows). However, this did not differ between control and ketoprofen groups (25.8% and 43.3%, respectively; p = 0.149). Similarly, the proportion of sows with a prolonged farrowing duration (>300 min) did not differ between control and ketoprofen groups (32.3% and 26.7%, respectively; p = 0.632).

### Sow characteristics

Table 2 shows the reproductive performance of sows treated with ketoprofen postpartum in comparison with control sows. The reproductive performance of sows between control and ketoprofen groups did not differ significantly (p>0.05). However, the proportion of stillborn piglets per litter was relatively high in both groups (Table 2). Of all sows (n = 61), fever was detected in 33 sows (54.1%) during the first 3 days postpartum. The proportions of sows that had fever on days 1, 2, and 3 postpartum were 9.8%, 34.4%, and 26.2%, respectively. The incidence of sows that had fever at days 1, 2, and 3 postpartum in the sows treated with ketoprofen compared with the control is presented in Table 3. During the first 3 days postpartum, the incidence of sows that had fever did not differ between ketoprofen and control groups (56.7% and 51.6%, respectively; p = 0.692). However, across groups, the incidence of sows that had fever at day 2 postpartum was higher in sows with a prolonged farrowing duration (>300 min) compared to sows with a normal farrowing duration (22.2% vs 4.7%, respectively; p = 0.036). Abnormal vaginal discharge during the first 3 days postpartum was detected in 50.8% (31/61 sows) of the sows. The incidence of abnormal vaginal discharge did not differ between control and ketoprofen groups (Table 2). However, across groups, the sows that had a prolonged farrowing duration had a higher incidence of abnormal vaginal discharge than those that had a normal farrowing duration (77.8% and 39.5%; p = 0.006). Nevertheless, the incidence of abnormal vaginal discharge did not differ significantly between the sows that required farrowing assistant and those that did not require farrowing assistance (61.9% and 45.0%, respectively; p = 0.209). The average daily feed intake of sows during the first 4 days postpartum was 3.45±0.06 and 3.52±0.07 kg in control and ketoprofen groups, respectively (p = 0.461).

### Piglet characteristics

On average, body weight at birth of the live-born piglets was 1.32±0.32 kg (Table 1). Colostrum intake of the piglets varied considerably from 5.5 to 991.4 g (Table 1). Of all the piglets, 20.8% had inadequate colostrum intake. The proportion of piglets that had inadequate colostrum intake was 71.3%, 22.6%, and 5.4% in the piglets with low, moderate and high birth weights, respectively (p<0.001). The colostrum intake per kg of the piglet birth weight averaged 363.5±85.8 g/kg and varied among piglets from 2.9 to 1041.0 g/kg. The colostrum intake and colostrum intake per kg of body weight of piglets with low, moderate and high birth weight in the sows treated with ketoprofen postpartum compared with the control group is presented in Table 4. Regardless of the piglet birth weight, the colostrum intake of piglets per kg of body weight did not differ significantly between control and ketoprofen groups (313.2±7.6 and 313.0±7.9 g/kg, respectively; p = 0.984). Piglets with low birth weight had a lower colostrum intake per kg of body weight than piglets with moderate birth weight (301.4±8.7 and 324.9±6.9 g/kg, respectively; p = 0.009) but did not differ significantly compared to the piglets with high birth weight (312.9±5.7 g/kg; p = 0.190). Across groups, 8.8% of the live-born piglets died within 3 days of postnatal life (Table 1). The piglet mortality rate did not differ between control and ketoprofen groups (9.0% and 8.5%, respectively; p = 0.808). Likewise, the colostrum intake and body weight at birth of the live-born piglets did not differ between control and ketoprofen groups (Table 2). Interestingly, the mortality rate during the first 3 days of postnatal life in the piglets that had adequate colostrum intake was lower than those in piglets with inadequate colostrum intake (3.2% vs 15.2%, respectively; p<0.001). Across groups, piglet mortality rates were 24.8%, 7.2%, and 4.7% in piglets with low, moderate and high birth weights, respectively (p<0.001). Table 4 shows the piglet mortality rates during the first 3 days of postnatal life in those with low, moderate and high birth weight in the ketoprofen group compared with the control group. The piglet mortality rate was significantly associated with birth weight but did not differ significantly between treatments (Table 4).

### Colostrum yield and immunoglobulin G

The average colostrum yield of sows was 5.35±0.19 kg and 5.42±0.19 kg in control and ketoprofen groups, respectively (p = 0.808). Colostrum yield of sow parity numbers 3 to 5 was higher than that of sow parity numbers 6 to 9 (5.73±0.19 and 5.04±0.19 kg, respectively; p = 0.012). The colostrum yield of sow parity numbers 3 to 5 and 6 to 9 treated with ketoprofen postpartum compared with the control is presented in Table 5. In the ketoprofen group, the colostrum yield of sow parity numbers 3 to 5 was higher than that of sow parity numbers 6 to 9 (Table 5). The colostrum yield of multiparous sows was positively correlated with the number of piglets born alive per litter (r = 0.521; p<0.001) and the rectal temperature during the first 3 days postpartum (r = 0.317; p = 0.013). On the other hand, the colostrum yield of multiparous sows was negatively correlated with the percentage of stillborn piglets per litter (r = −0.379; p = 0.003). Other variables, including the total number of piglets born per litter, the percentage of mummified fetuses per litter and farrowing duration, were not correlated with colostrum yield of multiparous sows (Table 6).

Across groups, colostrum IgG in the sows averaged 41.6± 14.4 g/L (range, 15.7 to 98.7). The Brix value of the sow colostrum averaged 26.6%±2.7% (range, 20.1 to 34.5), and colostrum IgG was significantly correlated with the Brix value (r = 0.492; p<0.001; n = 61). Colostrum IgG did not differ significantly between control and ketoprofen groups (41.5 ±2.6 and 41.7±2.7 g/L, respectively; p = 0.956). Likewise, the Brix values did not differ between control and ketoprofen groups (26.3%±0.5% and 26.8%±0.5%, respectively; p = 0.520). The colostrum IgG and Brix value were not correlated with farrowing duration, rectal temperature and litter traits (Table 6).

## DISCUSSION

The present study demonstrates the efficacy of ketoprofen treatment in postpartum sows to control the incidence of post-parturient disorders compared to the conventional herd protocol. In the present study, the NSAIDs routinely used according to the herd veterinarian recommendation was tolfenamic acid. According to the EU regulation, the anti-inflammatory drugs registered for oral administration are sodium salicylic acid, paracetamol and ketoprofen, whereas the anti-inflammatory drugs registered for parenteral route include meloxicam, flunixin meglumine, tolfenamic acid, and ketoprofen [11,27]. Therefore, ketoprofen is the only one anti-inflammatory drug that can be used for either oral or parenteral administration. In the present study, only parenteral administration of ketoprofen was applied, demonstrating that administration of ketoprofen in postpartum sows for 2 days can control the evidence of post-parturient disorders in sows as effectively as the use of tolfenamic acid. Indeed, two sows (6.6%) in the ketoprofen group and four sows (12.9%) in the control groups required the third treatment. This indicates that, in practice, the use of anti-inflammatory drugs in postpartum sows should be considered individually, and additional treatment should be applied in some sows that had severe postpartum illness. The present study demonstrated that administration of ketoprofen intramuscularly at the recommended dose for 2 to 3 times postpartum can effectively alleviate pain caused by a prolonged parturition process. Thus, this can improve the sow ability to initiate lactation properly and provide sufficient amount of colostrum to their offspring during the first 24 h after farrowing. Ketoprofen is an NSAIDs that has analgesic, antipyretic and anti-inflammatory properties [13]. The maximum level of ketoprofen in pigs can be achieved within 1 h after intramuscular administration [13]. Moreover, the analgesic effect of ketoprofen persists for 12 to 24 h after intramuscular administration in pigs [14]. Therefore, repeated administration of ketoprofen every 12–24 h should be considered. In the previous study, a dose of 6.0 mg of ketoprofen/kg improved the analgesic property of the drug compared to the recommended dose of 3.0 mg/kg. This indicates that a doubling of the marketed dose in pigs may provide a clinically relevant increase in the analgesic effect [14]. However, to increase the dose of ketoprofen in sows, further studies on its side effects should be performed.

In the present study, the incidence of PDS was relatively low in both groups; the incidence of PDS in the sows treated with ketoprofen was 3.0% lower than that in the control sows (i.e., 6.7% vs 9.7%, respectively). Nevertheless, this difference was not statistically significant, indicating that administration of either ketoprofen or tolfenamic acid can control the evidence of PDS in multiparous sows under tropical conditions. An earlier study has demonstrated that a single intramuscular administration of ketoprofen to sows within 12 h after farrowing can decrease rectal temperature during 12 to 48 h postpartum compared to the non-treated group [27]. The diagnosis of PDS in sows is not easy and is usually based on a combination of increased body temperature of the sow (>39.4°C) during the first 12 to 18 h postpartum and clinical signs [27]. In the present study, PDS was mainly defined based on the appearance of the udder during the first 3 days postpartum. Postpartum dysgalactia syndrome is assessed according to the absence or presence of inflammation (reddening and swelling) and/or agalactia of at least one teat of the udder [28]. Thus, some sows that had high fever without any appearance of udder inflammation were not included. In the previous study, PDS was detected in 7.5% to 18.5% of sows during the first 3 days postpartum [29]. Constipation on the farrowing day is one of the risk factors associated with the occurrence of PDS in sows [29]. However, in the present study, the faecal score did not differ significantly between groups, indicating that to effectively control the incidence of PDS, both the use of anti-inflammatory drugs and management to reduce risk factors should be performed together.

The limitation of the present study is that negative control sows (i.e., the postpartum sows not treated with any anti-inflammatory drug) were not included. This is due to the fact that most of the sows, generally, need antipyretic, analgesic and anti-inflammatory medication during the postpartum period. When omitting this medication protocol, the sows might suffer from postpartum illness, which may lead to severe health problems. This may subsequently also cause high mortality rates in the suckling piglets. In practice, most veterinarians in commercial swine breeding herds in Thailand generally recommend medication of postpartum sows, using either steroidal or NSAIDs. In the previous study, even though anti-inflammatory medication was performed, fever was still detected in 93.7% of primiparous sows, 52.6% of sow parity numbers 2 to 4 and 47.6% of sow parity numbers 5 to 7 [4]. Likewise, in the present study, fever was still detected in 54.1% of the multiparous sows during the first 3 days postpartum. The occurrence of fever in postpartum sows is associated with various factors such as farrowing duration, constipation and type of anti-inflammatory drug. Tummaruk and Sang-Gassanee [4] found that the percentage of sows with fever increased from 40% to 100% when the farrowing duration increased from <2 to 4–8 h. In addition, the incidence of sows with fever on the first day postpartum was two times higher in sows with constipation than in sows with normal faeces (36.2% vs 16.7%) [29]. Interestingly, the use of flunixin meglumine after parturition in sows reduced the percentage of sows with fever from 61.3% to 22.6% within 2 days postpartum, whereas the percentage of sows with fever was not decreased in sows treated with dipyrone [4]. This indicates that the type of anti-inflammatory drug used in postpartum sows might play an important role in the incidence of post-parturient disorders in sows under tropical conditions. In Spain, ketoprofen has been tested in 753 sows from 15 commercial herds in comparison with untreated sows, and pre-weaning mortality of the piglets was reduced from 10.2% to 8.4% in the ketoprofen-treated group [19]. In the United Kingdom, a previous study has demonstrated the impacts of ketoprofen post-farrowing by intramuscular injection at 1.5 h after the last piglet birth on sow feed intake, nursing behavior and piglet performance in 24 primiparous and 32 multiparous sows [27]. However, the previous study cannot demonstrate significant benefits of ketoprofen regarding colostrum IgG concentration and feed consumption of sows during the first week postpartum [27]. Similarly, in the present study, neither colostrum IgG nor feed consumption of sows differed significantly between ketoprofen and control groups, and the estimated colostrum consumption of piglets was also similar between ketoprofen and control groups.

In general, sows must recover quickly after farrowing to allow their offspring access to functional teats as soon as possible after birth to consume colostrum. Earlier studies have demonstrated that colostrum consumption of the newborn piglets significantly influences their survival rate until weaning [25]. However, under field conditions, up to 26.6% of the piglets had inadequate colostrum intake [24]. Similarly, in the current study, 20.8% of the piglets had inadequate colostrum intake. This indicates that intensive care during the first 24 h postpartum must be improved to minimize illness of sows and thus enhance their lactation capacity. In the present study, colostrum intake of piglets did not differ significantly between control and ketoprofen groups. However, 23.6% of piglets in the control group and 17.8% of piglets in the ketoprofen group showed inadequate colostrum intake. Indeed, most of the piglets that had inadequate colostrum intake were those with a birth weight below 1.0 kg. In the present study, up to 71.3% of the piglets that had a birth weight of less than 1.0 kg had inadequate colostrum consumption, whereas only 5.4% of the piglets that had a birth weight ≥1.30 kg had inadequate colostrum consumption. This indicates that the care of small piglets is also important to improve their ability to access teats and consume adequate amounts of colostrum.

In the present study, although the NSAIDs were used continuously in postpartum sows, fever was still detected in postpartum multiparous sows at least once during the first 3 days postpartum. This indicates that not only the continuous use of effective anti-inflammatory drugs but also some other medications (e.g., antibiotics and vitamins) and proper management strategies for postpartum sows should be considered [30]. Tummaruk [22] demonstrated that intravenous supportive treatment with amino acids and vitamins in sows can improve their appetite during the first 2 days post-partum. Vila and Tummaruk [30] suggested that the attitude and skills of a stockperson play an important role in the piglet survival rate postpartum. Stockpersons with a positive attitude and gentle handling of newborn piglets may positively impact the emotional responses of the piglets to humans and thus improve animal wellbeing [30]. These findings indicate that postpartum care of sows and their offspring is one of the most important issues to be addressed in the modern swine industry to minimize the incidence of post-parturient disorders in sows and thus obtain improved piglet survival rates.

In conclusion, administration of ketoprofen in postpartum sows for 2 days can control the evidence of post-parturient disorders in sows as effectively as the use of tolfenamic acid. No negative impacts of ketoprofen on sow colostrum yield, piglet colostrum intake and piglet mortality were detected. Therefore, ketoprofen can be recommended as an alternative NSAIDs used in postpartum sows.

## Figures and Tables

**Table 1:** Descriptive statistics on sow characteristics, reproductive performance, farrowing duration (min), colostrum qualification, post-parturient disorders and piglet characteristics in Danish Landrace×Yorkshire crossbred multiparous sows

Variable	Mean±SD	Range
Sow characteristics (n = 61)		
Parity number	5.5±1.6	3–9
Gestation length (d)	116.2±1.2	113–119
Farrowing duration (min)	277.8±164.4	53–948
Total number of piglets born per litter	15.9±3.0	7–22
Number of piglets born alive per litter	13.7±2.7	7–18
Stillborn piglets per litter (%)	9.1	-
Mummified fetuses per litter (%)	3.8	-
Colostrum yield (kg)	5.39±1.07	2.68–7.75
Colostrum Iimmunoglobulin G (g/L)	41.6±14.4	15.7–98.7
Brix value (%)	26.6±2.7	20.1–34.5
Post-parturient disorders		
Fever (%)	54.1	-
Postpartum dysgalactia syndrome (%)	8.2	-
Abnormal vaginal discharge (%)	50.8	-
Fecal score	2.83±0.49	0–5
Piglet characteristics (n = 833)		
Body weight at birth (kg)	1.32±0.32	0.26–2.60
Colostrum intake (g)	414.1±139.4	5.5–991.4
Colostrum intake per kg of body weight (g/kg)	311.9±85.8	2.9–1,041
Piglets that had colostrum intake <300 g (%)	20.8	-
Mortality rate during the first 3 days of life (%)	8.8	-

SD, standard deviation.

**Table 2:** Sow characteristics, reproductive performance, farrowing duration (min), colostrum qualification, post-parturient disorders and piglet characteristics in sows treated with ketoprofen postpartum compared with the conventional protocol for the herd (control) (least-square means±SEM)

Variables	Control	Ketoprofen	p-value
Number of sows	31	30	-
Sow characteristics			
Parity number (mean±SD)	5.4±1.7	5.7±1.5	-
Gestation length (d)	116.2±0.2	116.1±0.2	0.849
Total number of piglets born per litter	16.0±0.6	15.7±0.6	0.750
Number of piglets born alive per litter	14.0±0.5	13.3±0.5	0.309
Stillborn piglets per litter (%)	8.2	10.2	0.461
Mummified fetuses per litter (%)	3.6	4.0	0.818
Farrowing duration (min)	294.1±29.8	261.5±31.5	0.456
Sow daily feed intake during the first 4 days postpartum (kg/d)	3.45±0.06	3.52±0.07	0.461
Colostrum			
Colostrum yield (kg)	5.35±0.19	5.42±0.19	0.808
Colostrum IgG (g/L)	41.5±2.6	41.7±2.7	0.956
Brix value (%)	26.4±0.5	26.8±0.5	0.520
Post-parturient disorders			
Fever (%)	51.6	56.7	0.692
Postpartum dysgalactia syndrome (%)	9.7	6.7	0.668
Abnormal vaginal discharge (%)	48.4	53.3	0.699
Fecal score	2.75±0.62	2.91±0.32	0.584
Piglet characteristics			
Number of piglets	434	399	
Body weight at birth (kg)	1.32±0.03	1.33±0.03	0.913
Colostrum intake (g)	378.5±10.2	373.5±10.6	0.736
Colostrum intake per kg of body weight (g/kg)	313.2±7.6	313.0±7.9	0.984
Piglets that had colostrum intake <300 g (%)	23.6	17.8	0.395
Mortality rate during the first 3 days of life (%)	9.0	8.5	0.808

SEM, standard error of the mean; IgG, immunoglobulin G.

**Table 3:** Incidence of multiparous sows that had high fever (i.e., rectal temperature ≥39.5°C) at 1, 2, and 3 days postpartum in the sows treated with ketoprofen postpartum compared with those treated according to the conventional herd protocol (control)

Days after farrowing	Control	Ketoprofen	p-value
Day 1	16.1^a^	3.3^A^	0.093
Day 2	32.3^a^	36.7^B^	0.717
Day 3	19.4^a^	33.3^B^	0.215

a, A,B Different superscripts within column differ significantly (p<0.05).

**Table 4:** Colostrum intake (g), colostrum intake per kg of body weight (g/kg) and mortality rate during the first 3 days of postnatal life in piglets with low (<1.0 kg), moderate (1.0 to 1.29 kg) and high (>1.3 kg) birth weight in Danish Landrace×Yorkshire multiparous sows that were treated with ketoprofen postpartum compared those treated according to the conventional herd protocol (control) (least-square means±SEM)

Variables	Control	Ketoprofen	p-value
Low birth weight piglets			
Colostrum intake (g)	270.8±15.7	252.2±16.9	0.420
Colostrum intake per kg of body weight (g/kg)	306.3±11.9	296.6±12.8	0.581
Mortality rate (%)	21.3	29.0	0.358
Moderate birth weight piglets			
Colostrum intake (g)	380.3±12.6	383.4±13.2	0.860
Colostrum intake per kg of body weight (g/kg)	322.9±9.5	326.9±9.9	0.770
Mortality rate (%)	7.4	6.9	0.911
High birth weight piglets			
Colostrum intake (g)	484.5±7.9	485.0±11.2	0.972
Colostrum intake per kg of body weight (g/kg)	310.4±8.4	315.4±8.4	0.676
Mortality rate (%)	5.8	3.6	0.315

SEM, standard error of the mean.

**Table 5:** Colostrum yield (kg) in Danish Landrace×Yorkshire sows parity numbers 3–5 and 6–9 treated with ketoprofen postpartum compared with the conventional herd protocol (control) (least-square means±SEM)

Group of sows	Control	Ketoprofen	p-value
Parity numbers 3–5	5.65±0.26^a^	5.81±0.27^A^	0.654
Parity numbers 6–9	5.06±0.27^a^	5.02±0.27^B^	0.919

SEM, standard error of the mean.

a, A,B Different superscripts within column differ significantly (p<0.05).

**Table 6:** Correlation between colostrum yield (kg), colostrum IgG (g/L) and Brix value (%) of sow colostrum and farrowing duration (min) and reproductive traits in multiparous sows (correlation coefficient)

Variables	Colostrum yield	Colostrum IgG	Brix value^1)^
Total number of piglets born per litter	NS	NS	NS
Number of piglets born alive per litter	0.510^***^	NS	NS
Stillborn piglets per litter	−0.504^***^	NS	NS
Mummified fetuses per litter	NS	NS	NS
Farrowing duration	NS	NS	NS
Maximum rectal temperature postpartum	0.268^*^	NS	NS

1) The measurement of Brix value of fresh colostrum in sows has been validated to be use as a rapid method for estimating IgG concentration of colostrum [10].

* p<0.05,

** p<0.01,

*** p<0.001,

NS, not significant.
